# Gap filling in long-term AOD time series using Iterative Singular Spectrum Analysis at a tropical AERONET site

**DOI:** 10.1007/s10661-026-16067-6

**Published:** 2026-10-30

**Authors:** Tiago Schmidt, Rafael da Silva Palácios, Amaury de Souza, Leone Francisco Amorim Curado

**Affiliations:** 1 Department of Teaching, Federal Institute of Education, Science and Technology of Mato Grosso, Rua das Avencas, Sinop, 78550-178 Mato Grosso Brazil; 2 Graduate Program in Environmental Physics, Federal University of Mato Grosso, Avenida Fernando Corrêa da Costa, Cuiabá, 78060-900 Mato Grosso Brazil; 3 Institute of Geosciences, Federal University of Pará, Rua Augusto Corrêa, Belém, 66075-110 Pará Brazil; 4 Physics Department, Federal University of Mato Grosso do Sul, Avenida Costa e Silva, Campo Grande, 79070-900 Mato Grosso do Sul Brazil

**Keywords:** Aerosol optical depth, AERONET, Gap filling, Singular Spectrum Analysis

## Abstract

Aerosol optical depth (AOD) is widely used in studies related to atmospheric monitoring, climate variability, and air quality, with the Aerosol RObotic NETwork (AERONET) providing high-quality records. However, AERONET AOD time series frequently contain observational gaps associated with cloud cover, especially in tropical regions characterized by strong hydrological seasonality. In this study, the performance of Iterative Singular Spectrum Analysis (ISSA) was evaluated for gap-filling an incomplete AOD time series from the AERONET Cuiabá-Miranda station (AOD-AERONET) in the Central-West region of Brazil. Daily AOD-AERONET data between 2005 and 2024 were used, with approximately 47% of the daily observations identified as gaps and showing a predominantly seasonal distribution. The application of ISSA to the gap-filling of the AOD series involved the evaluation of different initialization and cross-validation strategies using model performance metrics, as well as different reconstruction parameters. The results showed that the climatological initialization, based on the Day of the Year (DOY) mean, outperformed the global-mean initialization, producing smaller errors and greater temporal consistency of the reconstruction, and that the cross-validation design plays an important role in the selection of optimal parameters.

## Introduction

Atmospheric aerosols influence the terrestrial radiative balance, regional climate variability, cloud formation, and air quality, being associated with adverse effects on human health (Couto et al., 2020; IPCC, 2021; Vos et al., 2020). Among the main parameters used to characterize atmospheric particulate matter, aerosol optical depth (AOD) stands out as a widely used metric in environmental, climate, and remote sensing studies (Franco et al., 2024; Palácios et al., 2020), serving as an important predictor in models of atmospheric particulate matter concentration (Liu et al., 2009; Zhang et al., 2018). In tropical regions of South America, especially in the Brazilian Cerrado and the Amazonian surroundings, AOD exhibits strong seasonality associated with the hydrological regime, which is closely associated with the occurrence of biomass burning, concentrated in the dry season, as well as precipitation and cloud cover, concentrated during the wet season (Artaxo et al., 2013, 2022; Franco et al., 2024; Palácios et al., 2020, 2022).

AOD monitoring is performed in a continuous and globally spatialized manner by the AERONET (Aerosol Robotic Network) ground-based spectrophotometer network, which constitutes an important observational reference for atmospheric monitoring and for the validation of orbital AOD products by direct observation or reanalysis models (Holben et al., 1998; Júnior et al., 2025; Rudke et al., 2023; Tuna Tuygun & Elbir, 2024). However, long-term AOD-AERONET time series frequently present observational gaps primarily related to the specific atmospheric condition of persistent cloud cover, which hampers the acquisition of valid measurements of direct solar radiation (Olcese et al., 2015). In tropical regions, these missing records tend to exhibit a seasonal pattern, following periods of increased cloudiness and precipitation, potentially occurring for several consecutive days, which generates continuous and seasonally structured temporal gaps. This characteristic imposes additional challenges to time series reconstruction, since conventional cross-validation approaches based solely on the random removal of observations may not adequately represent the actual gap-filling conditions encountered in operational applications.

These continuity gaps in the time series data points can compromise statistical analysis and estimates of the variability and trend of the series, due to distortions, biases, and the limitation of available information (Filho & Lima, 2016; Olcese et al., 2015; Rekapalli & Tiwari, 2015; Yang & Buu, 2025). Time series gap filling can reduce these problems (Yi & Sneeuw, 2021) and, specifically in the case of AOD series, improve the performance of atmospheric radiative forcing or air quality models associated with aerosols (Parmar et al., 2024; Zhang et al., 2018).

Singular Spectrum Analysis (SSA) is a *model-free* method of time series decomposition and reconstruction, which aims at the analysis of the series by empirical orthogonal functions (EOF) and elementary components, which can be interpreted as trend, seasonality, other periodic variations, and noise (Golyandina, 2020; Zhigljavsky & Golyandina, 2010). In SSA, only intrinsic properties of the series are considered, preserving its internal processes. These characteristics can be explored in the reconstruction of incomplete series, adding reliability in relation to interpolation filling methods (Rekapalli & Tiwari, 2015).

The use of SSA as a gap-filling method in time series is explored through different approaches in the literature, either via iterative algorithms for the determination of optimal reconstruction parameters (Golyandina, 2020; Kondrashov & Ghil, 2006; Musial et al., 2011; Rekapalli & Tiwari, 2015; Yi & Sneeuw, 2021) or through other approaches focused on the analysis of the properties of elementary components or the sequential reconstruction of residuals (Filho & Lima, 2016; Golyandina, 2010; Golyandina & Osipov, 2007; Hassani et al., 2019; Yang & Buu, 2025).

**Fig. 1:**
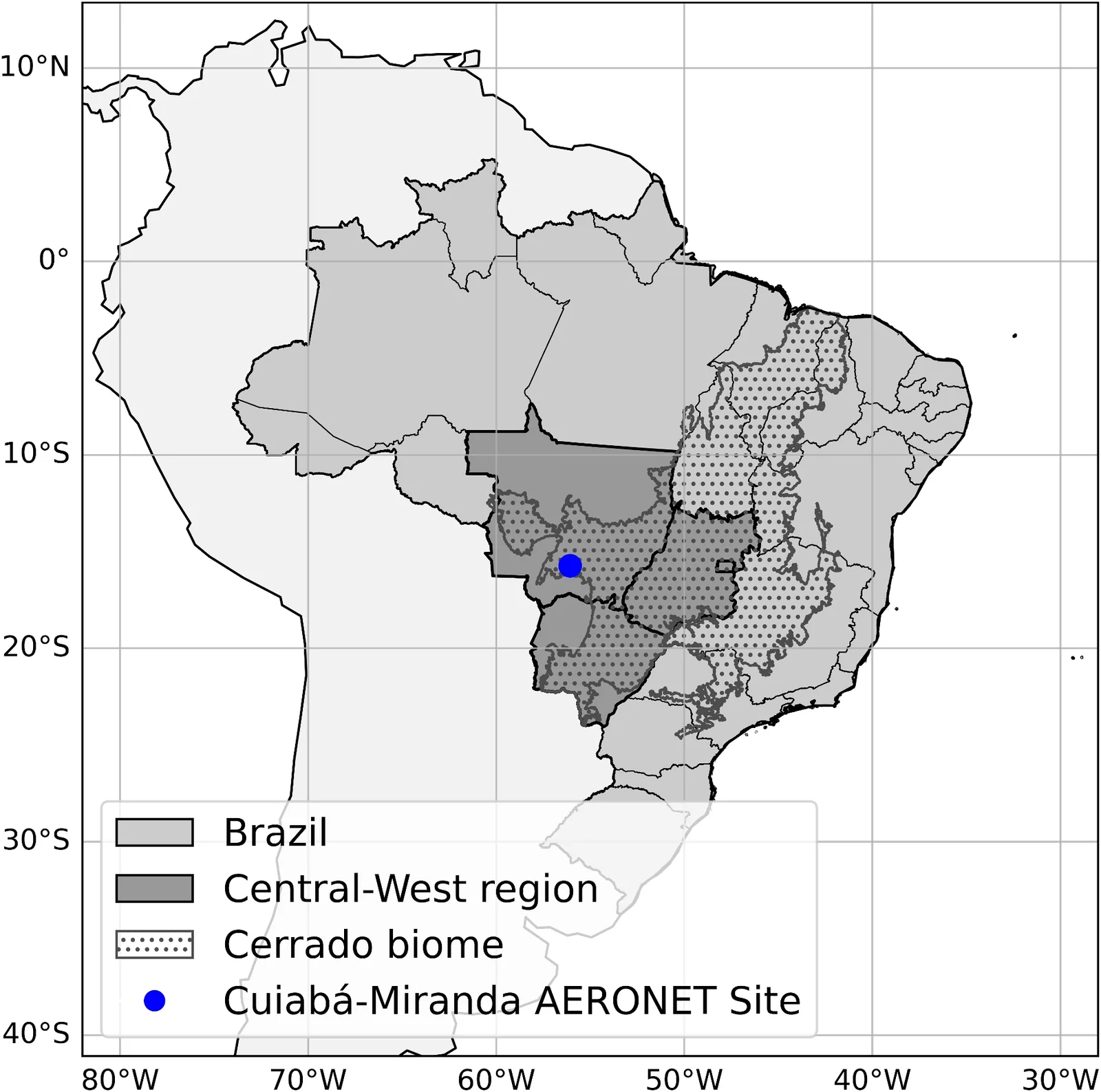
Location of the AERONET Cuiabá-Miranda site, in the Brazilian Central-West in a region of the Cerrado biome

In particular, Iterative Singular Spectrum Analysis (ISSA), as a gap-filling method for incomplete time series (Kondrashov & Ghil, 2006), yields superior or comparable results to interpolation methods for different missing data configurations, including long intervals. It is characterized by its robustness and flexibility in the presence of periodic components, with the capability of maintaining variation patterns across different applications, particularly in global geophysical time series with artificially created gaps, or in simulations on synthetic time series (Kondrashov & Ghil, 2006; Musial et al., 2011; Rekapalli & Tiwari, 2015; Yi & Sneeuw, 2021).

Despite advances achieved with different SSA-based approaches for the gap-filling reconstruction of incomplete environmental time series, studies focused on filling AOD-AERONET time series, especially in tropical regions, using exclusively intrinsic information from the time series itself, remain scarce. In this context, the main innovation of this study consists of the systematic application of Iterative Singular Spectrum Analysis (ISSA) for the gap filling of a long-term AOD-AERONET time series in a tropical environment, which presents naturally occurring observational gaps that are strongly associated with local environmental characteristics, particularly the pronounced climatic seasonality, resulting in high data variability and a non-random distribution of the observational gaps. The time series reconstruction with estimation of values on days with missing data was performed without using auxiliary variables such as orbital remote sensing products or external atmospheric models, exploring only the temporal dependence information contained within the series itself. Additionally, different initialization and cross-validation strategies were evaluated, including approaches capable of better representing systematic patterns and persistent temporal gaps, typical of atmospheric records in tropical regions.

## Materials and methods

### Study area and data

The data used in this study were obtained from observations at the AERONET Cuiabá-Miranda site, with coordinates ($15.73091^{\circ }$ S, $56.07086^{\circ }$ W) (Fig. 1), located in the municipality of Cuiabá, state of Mato Grosso, Brazil. The region is situated in a transition area between the Cerrado and Pantanal biomes, characterized by the strong climate seasonality typical of the Brazilian Central-West, with well-defined wet and dry seasons, an Aw climate according to the Köppen classification. It features a well-defined rainy period between October and April, with an average of 15 rainy days per month Machado et al. (2015), and is further characterized by the occurrence of biomass burning during the dry season (Palácios et al., 2018, 2020).

Daily Aerosol Optical Depth data at a wavelength of 500 nm were used, obtained from the AERONET website (AOD-AERONET), with processing level 2.0, which represents the highest level of quality and data control provided by the network. This level includes calibrations with corrections for local factors and cloud screening (Holben et al., 1998; Palácios et al., 2022). This protective layer, while increasing observational reliability, also significantly reduces the amount of valid measurements available, especially during periods of high cloudiness Olcese et al. (2015), which implies an increase in the proportion of observational gaps in medium- or long-term time series.

The records from this site have been available since 2001, and the overall period of the analyzed data extends from August 2005 to August 2024, totaling 6971 daily observations (valid or not) referring to the average over the valid observations for each day. Days with no valid observations were considered observational gaps in the time series. It was verified that the resulting data series presented a high gap rate, concentrated mainly in the rainy months, when cloud persistence reduces the availability of valid observations, obtained by direct radiation captured in the sun photometer. Over the period of 19 years and one month, 3253 daily observations, which corresponds to approximately 47% of the total, resulted in missing data, with a strongly seasonal temporal distribution and in different configurations. These characteristics make the series a particularly suitable object for the evaluation of ISSA as a gap-filling method for observational gaps in incomplete atmospheric records under climate variability conditions of tropical regions.

### Singular Spectrum Analysis (SSA)

SSA is a non-parametric method for discrete time series analysis, and its use is not associated with specific behaviors regarding data distribution. It enables exploring the dynamics of the underlying system of the time series from a single record of it Elsner and Tsonis (1996). The method has been used in environmental and geophysical studies for trend analysis, separation of seasonal and oscillatory components, noise reduction, and the gap filling of incomplete time series (Golyandina, 2020; Kondrashov & Ghil, 2006; Palácios et al., 2016; Rekapalli & Tiwari, 2015; Yang et al., 2022; Zhigljavsky & Golyandina, 2010).

Basic SSA (Golyandina, 2010, 2020) is based on the spectral distribution of the singular values of the *trajectory matrix* defined by lagged vectors constructed from sliding windows over the time series $\boldsymbol{x} = (x_{1}, x_{2}, \dots x_{n})$ and on its respective singular vectors. The method consists of two stages, Decomposition and Reconstruction. Decomposition is divided into two steps: embedding and singular value decomposition (SVD). Reconstruction is divided into grouping and diagonal averaging reconstruction.

The *embedding* of the series into an *L*-dimensional vector space corresponds to mapping the series $\boldsymbol{x}$ into a matrix $\boldsymbol{M}\in \mathbb {R}^{L\times K}$, with rank $p\le min\lbrace L, K \rbrace$. Matrix $\boldsymbol{M}$ is referred to as the *Trajectory Matrix* and is fully determined by its column vectors $\boldsymbol{X}_j = (x_{j}, x_{j+1}, \dots x_{j+L-1})^{T},\, j=1, 2, \dots , K$ (Eq. 1), referred to as *L*-lagged vectors, which span the *trajectory space* of dimension *p*. These vectors carry information from $\boldsymbol{x}$, through *K* sliding windows of length *L*, as overlapping subseries of the original series.

1 $$\begin{aligned} \boldsymbol{M} = \begin{bmatrix} x_{1} & x_{2} & \dots & x_{K} \\ x_{2} & x_{3} & \dots & x_{K+1} \\ \vdots & \vdots & \ddots & \vdots \\ x_{L} & x_{L+1} & \dots & x_{L+K-1} \end{bmatrix} \end{aligned}$$

The matrix $\boldsymbol{M}$ is a Hankel matrix, with equal values along each anti-diagonal. The relationship between elements of $\boldsymbol{M}=[m_{ij}]_{LK}$ and the series points is $m_{ij} = x_{i+j-1}$ and the dimensional relationship between $\boldsymbol{M}$ and the series $\boldsymbol{x}$ is n=L+K-1. The window length *L* is a key parameter of the SSA method, as it influences its ability to capture temporal variability at different scales.

In the SVD step, the trajectory matrix is decomposed as in Eq. 2.

2 $$\begin{aligned} \boldsymbol{M}= &  \boldsymbol{U} \boldsymbol{\Sigma } \boldsymbol{V}^{T} \nonumber \\= &  \sum _{i=1}^{p} \sigma _i \boldsymbol{u}_{i} \boldsymbol{v}_{i}^{T} = \sum _{i=1}^{p}\boldsymbol{M}_{i} \end{aligned}$$

where $\boldsymbol{U} = (u_{ij})_{L\times L}$ and $\boldsymbol{V} = (v_{ij})_{K\times K}$ are orthogonal matrices whose columns are formed by the sets of orthonormal eigenvectors $\lbrace \boldsymbol{u}_l;\, l=1,\dots , L \rbrace$ and $\lbrace \boldsymbol{v}_l;\, l=1,\dots , K \rbrace$, of the symmetric matrices $\boldsymbol{M}\boldsymbol{M}^T$ and $\boldsymbol{M}^T\boldsymbol{M}$, respectively. The first *p* eigenvectors in each set are associated with the *p* nonzero eigenvalues $\lambda _l$, ordered such that $\lambda _1 \ge \lambda _2 \ge \dots \ge \lambda _p > 0$. The rectangular matrix $\boldsymbol{\Sigma }_{L\times K}$ has zero entries except on its main diagonal, where the first *p* entries correspond to the nonzero singular values of $\boldsymbol{M}$, $\sigma _l = \sqrt{\lambda _l}$. The vectors $\lbrace \boldsymbol{u}_l \rbrace$ for l=1,⋯,p span the *column space* of $\boldsymbol{M}$ (Golyandina, 2010), and are called *Empirical Orthogonal Functions (EOFs)* (Beckers & Rixen, 2003; Kondrashov & Ghil, 2006; Musial et al., 2011).

In Eq. 2, $\boldsymbol{M}$ is decomposed into *p* rank-one elementary matrices $\boldsymbol{M}_{i}$, defined by the respective *eigentriple* $(\sigma _i, \boldsymbol{u}_{i}, \boldsymbol{v}_{i} )$ and associated with an elementary component (EC) of $\boldsymbol{x}$, defined by the projection of the sliding windows onto the corresponding EOF, with the projection coefficients scaled by the corresponding $\sigma _i$. Thus, the EOFs associated with the d≤p largest singular values are those that carry the most information from the original series in the respective ECs. The value *d* is another important parameter of SSA, as it determines the amount of temporal variability retained when reconstructing the series represented by *x*, by specifying the number of dominant EOFs considered, while discarding noise or other unwanted patterns.

The *grouping* step is outlined according to the reconstruction objective, ranging from separating the trend from other patterns to simple filtering between signal and noise. It is performed on the *p* eigentriples associated with the nonzero singular values by partitioning the index set of the elementary matrices into *m* disjoint subsets $I_i$ such that $\displaystyle \bigcup _{i=1}^{m} I_i = \lbrace 1,\dots , p \rbrace$, and summing the elementary matrices over each $I_i$, as $\boldsymbol{M}_{I_i}=\sum _{j\in I_{i}} \boldsymbol{M}_j$. Thus, each group is indexed by $I_i, \, i=1, \dots , m$, and the grouped decomposition is denoted by $\boldsymbol{M} = \sum _{i=1}^{m}\boldsymbol{M}_{I_i}$. The reconstructed trajectory matrix $\widetilde{\boldsymbol{M}}$, obtained after selecting the groups to be retained, is given by Eq. 3.

3 $$\begin{aligned} \widetilde{\boldsymbol{M}} = \sum _{i=1}^{m'}\boldsymbol{M}_{I_i} \end{aligned}$$

where $m^{\prime }\leq m$, since the groups retained in the reconstruction form a subset of the total, potentially resulting in a lower-rank reconstructed trajectory matrix when $m^{\prime }<m$ (Rekapalli & Tiwari, 2015).

In the *diagonal averaging* step, each grouped matrix $\boldsymbol{M}_{I_i}$ is approximated by a Hankel matrix $\widetilde{\boldsymbol{M}}_{I_i}$ by replacing the elements along each anti-diagonal with their average. The resulting Hankel matrix is then mapped into a component series $\widetilde{\boldsymbol{x}}^{I_i}$ of $\boldsymbol{x}$. The reconstructed series is then given by Eq. 4.

4 $$\begin{aligned} \widetilde{\boldsymbol{x}} = \widetilde{\boldsymbol{x}}^{I_1} + \dots + \widetilde{\boldsymbol{x}}^{I_{m'}} \end{aligned}$$

If m=p, an *elementary grouping* is obtained (Golyandina, 2020), and each $\widetilde{\boldsymbol{x}}^{I_i}$ corresponds to an elementary component (EC) of the reconstructed series. When the reconstruction is based only on a selected number *d* of dominant eigentriples, the grouping can be defined with m=2, where $I_1 = \lbrace 1, \dots , d \rbrace$ represents the eigentriples retained for reconstruction, and $I_2 = \lbrace d+1, \dots , p \rbrace$ represents the discarded components. This grouping scheme is adopted in each successive reconstruction in the iterative gap-filling algorithm used in this study.

### Iterative SSA gap-filling algorithm

The reconstruction of the incomplete time series was performed using an iterative approach based on Singular Spectrum Analysis, hereafter referred to as ISSA (Iterative SSA), adapted from different proposals (Beckers & Rixen, 2003; Kondrashov & Ghil, 2006; Yi & Sneeuw, 2021). Iterative variants and other SSA-based gap-filling methods have been proposed in the literature (Golyandina & Osipov, 2007; Hassani et al., 2019; Yang & Buu, 2025; Yi & Sneeuw, 2021), demonstrating the versatility of SSA for gap-filling in incomplete time series.

The ISSA procedure consists of successive reconstructions of the time series using SSA, with iterative updating of the missing values at each reconstruction, using optimal *L* and *d* parameters, determined from cross-validation statistics.

**Fig. 2:**
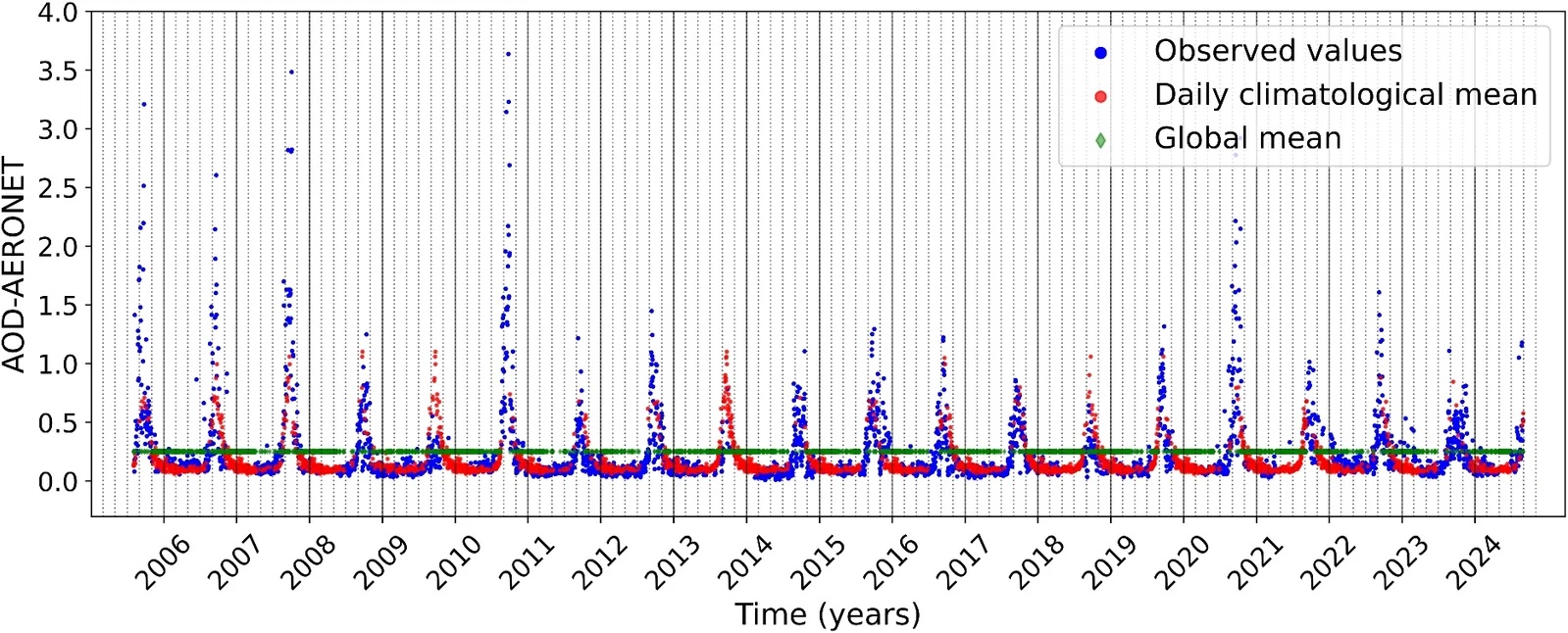
Gap-filling initialization using the global mean and the daily climatological mean. AOD values greater than 4 were clipped for better visualization

In principle, the missing values in the series were replaced by values estimated through an initialization procedure, allowing the construction of the trajectory matrix and the application of SVD. Each gap-filling reconstruction in ISSA consists of a sequence of reconstructions of the series for a given window length *L*, with the number of dominant EOFs retained in a single group increasing from 1 to *d*, while the missing values are updated at each iteration (Beckers & Rixen, 2003; Hassani et al., 2019; Kondrashov & Ghil, 2006; Kondrashov et al., 2010).

The algorithm was structured with a single iterative level of reconstruction and gap filling. This loop iterates over the quantity *d* of dominant EOFs used in successive single-group reconstructions. For each window length *L*, the first iteration involves reconstructing the initialized series $\boldsymbol{x}$, denoted by $\widetilde{\boldsymbol{x}}_0$, with d=1, and updating the values at the missing points with the corresponding reconstructed values, obtaining the series $\widetilde{\boldsymbol{x}}_1$. The procedure is repeated, but starting with $\widetilde{\boldsymbol{x}}_1$ and using d=2, resulting in the reconstructed and gap-filled series $\widetilde{\boldsymbol{x}}_2$. The process of cumulative reconstructions repeats until a predefined value of *d*.

The tested values of *L* were defined to represent different characteristic time scales of the series, including periodicities associated with seasonal variability and the temporal persistence of atmospheric data. For each tested *L*, *d* was varied in unit steps, resulting in a large number of (*L*, *d*) combinations.

#### Time series initialization strategies

In this study, different initialization strategies for the AOD series required for the first iteration of ISSA were evaluated. The first approach consisted of replacing the missing values with the global mean of the observed valid points. This practice is frequently cited in the literature, either through direct substitution or through centering the series around zero (Beckers & Rixen, 2003; Hassani et al., 2019; Kondrashov & Ghil, 2006; Kondrashov et al., 2010). Despite its computational simplicity, this strategy can lead to a poor assessment of reconstruction performance, given the amount of temporal information ignored in a strongly seasonal series (Schaefli & Gupta, 2007; Williams, 2025). Another issue is the production of unrealistic patterns of values and local temporal variability when the gap pattern is also seasonal or systematic Musial et al. (2011).

Another strategy evaluated was the *daily climatological mean*, calculated by averaging values for each day of the year over the entire period of the time series. This approach, based on the mean for each “Day of the Year” (DOY), resulted in greater temporal consistency in the AOD values and provided a richer reference for evaluating the efficiency of ISSA gap filling (Schaefli & Gupta, 2007).

Figure 2 clearly shows the temporal profile of the studied series, with evident annual seasonality, marked variability, and a seasonal concentration of observational gaps between October and March. It also illustrates how the two tested initialization approaches adapt to the temporal patterns of the series. The points initialized using the global mean form a temporal structure that is poorly consistent with the seasonal variability observed in the original series and with the expected lower AOD values during the rainy periods. In contrast, the initialization by the DOY-grouped mean provides a better visual fit to the temporal structure of the AOD-AERONET series.

The comparison of different initialization approaches aimed to evaluate the sensitivity of ISSA reconstruction to initial conditions, considering the high proportion of missing data and its non-random distribution over the years. The outcomes of the different initialization strategies were compared based on cross-validation metrics and optimal parameter combinations, as well as on the visual patterns of the resulting gap-filled series.

#### Cross-validation and statistical metrics

The performance of the gap-filling method, under different parameter settings and conditions, was evaluated by cross-validation, based on the insertion of artificial observational gaps at originally valid points in the time series (Kondrashov & Ghil, 2006). The cross-validation metrics allow the reconstruction parameters and initialization strategies to be evaluated, guiding the minimization of the global error in the final gap filling through the selection of optimal ISSA parameters (Golyandina, 2020; Kondrashov & Ghil, 2006; Kondrashov et al., 2010) for each initialization strategy.

For each combination of the parameters *L* and *d*, 40 iterative SSA reconstruction experiments were performed. In each experiment, a different random *validation set* of valid points from the original series $\boldsymbol{x}$ was removed and temporarily treated as gaps, and the reconstruction was performed on the resulting modified series $\boldsymbol{x}'$. For each $\boldsymbol{x}'$, whose set of valid points is a subset of that of the original series, the metrics were calculated exclusively over the corresponding validation set.

The formation of the validation sets considered the temporal distribution of observational gaps, seeking to represent conditions associated with the persistence of missing data during rainy periods, which result in a high concentration, or continuous gaps, of missing AOD records. Thus, although the validation sets were randomly defined, they followed criteria established for two approaches, applied separately. In the first, cross-validation was performed on *random points* within months with at least 50% missing data. In the second approach, aimed at reducing reconstruction patterns observed when gap filling was evaluated using random points, the validation sets were defined as *continuous gaps*, simulating gaps associated with persistent cloud cover and representing the most critical gap-filling situation for ISSA in terms of the configuration of missing points (Kondrashov & Ghil, 2006).

The gap-filling performance was evaluated using statistical metrics based on the error $e_i = x_{i, pred} - x_{i,obs}$ between the predicted value from the reconstruction, $x_{i, pred}$, and the corresponding observed value, $x_{i,obs}$, at each time step *i*. The metrics used include the *root mean square error* (RMSE), the *mean absolute error* (MAE), and the *mean error* or *bias* (Eq. 5).

5 $$\begin{aligned} \text {RMSE}&= \sqrt{\dfrac{1}{n}\sum _{i=1}^{n}e_i^2} \nonumber \\ \text {MAE}&= \dfrac{1}{n}\sum _{i=1}^{n}|e_i| \nonumber \\ \text {Bias}&= \dfrac{1}{n}\sum _{i=1}^{n}e_i \end{aligned}$$

While RMSE and MAE are indicated for evaluating accuracy or skill, bias allows for an assessment of the method’s tendency to overestimate or underestimate the validation values, and all of them can be used to compare the method across different series (Hallak & Pereira Filho, 2011; Williams, 2025; Yang & Buu, 2025).

**Fig. 3:**
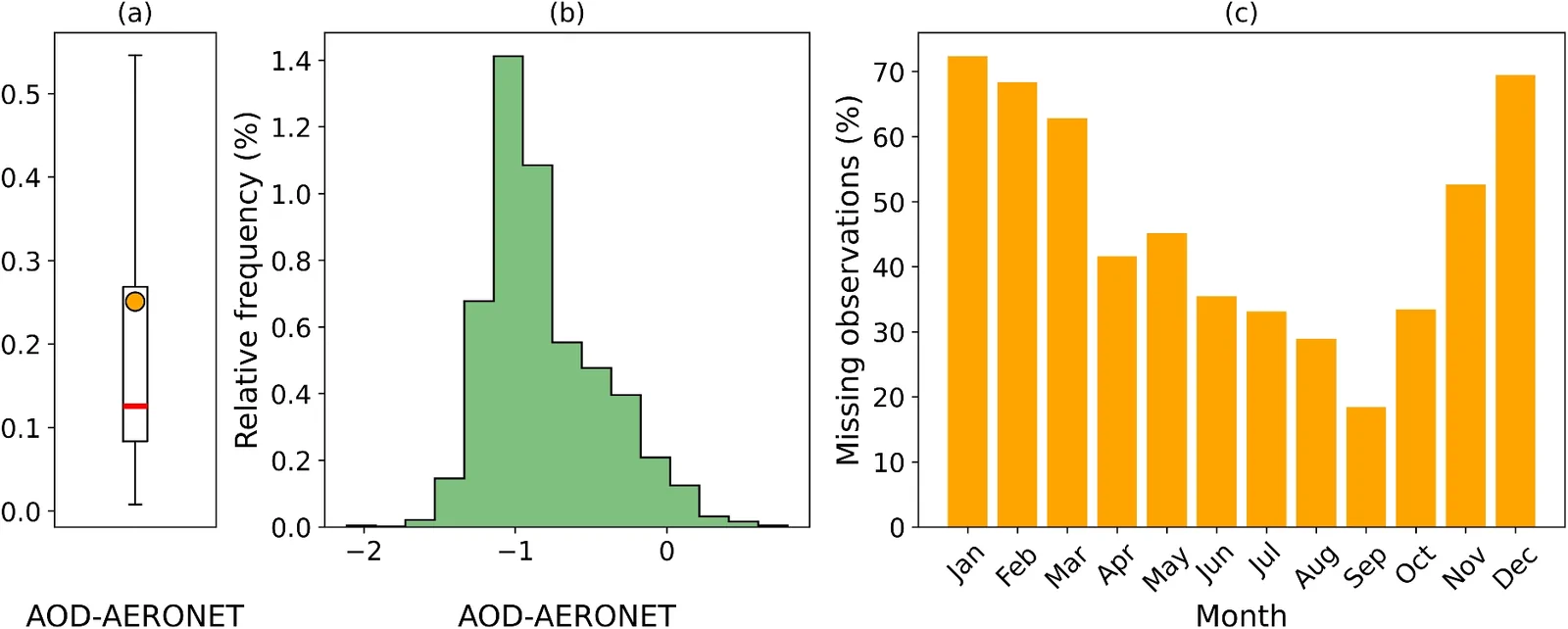
**a** Boxplot of valid observations over the study period. **b** Frequency distribution of decimal logarithm values of AOD-AERONET at valid observation points. **c** Average percentage of missing observations for each month of the year over the period from August 2005 to August 2024

In addition to these metrics, the *Modified Efficiency Coefficient* (MEC) (Eq. 6) was used, based on the *Nash-Sutcliffe Efficiency* (NSE) coefficient, which was developed for model parameter calibration and can quantify relative skill when compared to a simpler approach or model (Schaefli & Gupta, 2007; Williams, 2025), such as initialization gap-fillings, for example.

6 $$\begin{aligned} \text {MEC} = 1- \dfrac{\sum _{i=1}^{C} e_i^2 }{\sum _{i=1}^{C} \left( x_{i,obs} - x_{i,ref} \right) ^2} \end{aligned}$$

where *C* is the cardinality of the validation set, in this case C=30. Each $x_{i,pred}$ represents a value filled by the evaluated model within the validation set, while $x_{i,obs}$ and $x_{i,ref}$ represent the corresponding observed value and reference-model value at the same point, respectively. The MEC was employed to evaluate the relative performance of ISSA with respect to the series initialization strategies, with positive values indicating improved gap-filling performance and an upper bound of 1, corresponding to a perfect fit to the validation data (Schaefli & Gupta, 2007). Negative MEC values are possible and indicate poorer performance than the reference filling. The initial guesses based on both the DOY mean and the global mean were recalculated from the valid observations in each of the 40 cross-validation experiments after the corresponding validation set was created, thereby avoiding data leakage. By design, MEC values obtained from different initializations strategies cannot be directly compared.

The final cross-validation metrics for each combination of reconstruction parameters and each tested initialization strategy were estimated as the respective averages of the metrics across the experiments. The parameter pair (*L*, *d*) considered optimal was defined based on the joint analysis of the lowest RMSE, MAE, and bias values and the highest relative efficiency (MEC).

## Results and discussions

### Environmental properties of the variable and temporal structure of observational gaps

The AOD-AERONET series exhibited a strong positive skewed distribution, with a predominance of low values and the occurrence of extreme and seasonal events associated with the dry season and the intensification of regional biomass burning (Artaxo et al., 2022; Franco et al., 2024; Palácios et al., 2020). The boxplot of the series (Fig. 3a) highlights this asymmetry, with most observations lying below the mean (orange circle). The mean is close to the upper quartile and substantially higher than the median (red line), while the distribution also exhibits high dispersion. Extreme AOD values were not shown for visualization purposes, but can exceed the value of 6 in the dry season of some years.

The minimum and maximum values of the series are $x_{min} = 0.007725$ and $x_{max} = 6.268689$, respectively, with a mean value $\bar{x} = 0.250882$, a median $\tilde{x} = 0.125772$, and a standard deviation $\text {s}=0.361717$. The histogram of the decimal logarithm of AOD-AERONET (Fig. 3b) further illustrates the predominance of low AOD values, with a right tail associated with high aerosol loading events.

The AOD-AERONET time series presented a high proportion of observational gaps throughout the study period, with a strongly seasonal temporal distribution. Approximately 47% of the days did not have valid observations, highlighting the elevated observational constraints associated with atmospheric optical monitoring in humid tropical regions. The monthly distribution of the gaps showed a clear concentration during the wet season, particularly between November and March, with an average gap rate higher than 50%, reaching over 70% in January (Fig. 3c).

The seasonality observed in the gaps directly follows the regional hydrological cycle (Machado et al., 2015) associated with the cloud-cover patterns in the region. Since AERONET sun photometers depend on the observation of direct solar radiation, prolonged periods of cloudiness significantly reduce the availability of valid AOD measurements (Olcese et al., 2015). This non-random characteristic of the temporal distribution of observational gaps reflects a physical limitation of surface optical records, associated with the predominant atmospheric conditions in the study area.

The highest AOD values occurred mainly between August and October, coinciding with the period of maximum biomass-burning activity. On the other hand, during the wet season, AOD levels are much lower and are primarily due to local urban emissions (Franco et al., 2024). This profile of high temporal variability is characteristic of the state of Mato Grosso and also of the Amazon region and its surroundings, known as the “deforestation arc”, and reflects the alternation between long periods of low aerosol concentration and episodes of intense particle emission into the atmosphere (Franco et al., 2024; Palácios et al., 2018, 2022). September is the last month of the dry season (Biudes et al., 2015), and is also the month with the highest AOD peaks, a higher occurrence of fire spots (Palácios et al., 2018, 2022), and the lowest percentage of observational gaps (Fig. 3c).

The combination of the high temporal variability of AOD-AERONET, which increases the complexity of the time series, and the high rate of observational gaps, characterized by a seasonal and almost systematic pattern resulting from the temporal persistence of cloudy meteorological conditions, represents a significant challenge for temporal gap-filling methods (Kondrashov & Ghil, 2006; Musial et al., 2011).

These characteristics, combined with the capacity of SSA-based methods for filling continuous observational gaps (Filho & Lima, 2016; Kondrashov & Ghil, 2006; Musial et al., 2011; Rekapalli & Tiwari, 2015), make the AOD-AERONET series from the Cuiabá-Miranda station particularly suitable for evaluating the performance of ISSA under environmental conditions typical of seasonally humid tropical regions.

### Initialization strategies and temporal gap-filling efficiency

The initialization strategy used in each application of ISSA exerted a marked influence on the behavior of the data during the iterative reconstructions, especially due to the strong climatic seasonality observed in the study region, which imposes a systematic pattern on the temporal configuration of some of the observational gaps and contributes to the high variability of the AOD-AERONET series.

The use of the global mean as an initial guess for the gaps, as mentioned in the literature (Golyandina, 2010; Hassani et al., 2019; Kondrashov & Ghil, 2006), did not reproduce the characteristics of the series, disregarding the seasonality and resulting in the introduction of artificial patterns, particularly over the rainy months, where observational gaps occur more frequently, as evidenced by Figs. 2 and 3c. Due to the particularities of the series and the complexity added by the initialization using the global mean, different cross-validation approaches were tested in this case, leading to parameter choices with opposite characteristics.

The climatological initialization, on the other hand, allowed for the incorporation of underlying information on climate variability into the series, still in the initial stage of the ISSA application for gap-filling reconstruction, reproducing the variability of the observed values more realistically. The observed differences between the initialization strategies regarding their estimation capabilities in representing the dominant climatic and environmental structure evidenced that this is an important stage of ISSA as a method based solely on time-series data.

The results indicated that the climate dynamics underlying the presence of particulate matter and the physical capability of sun photometers to register AOD values played an important role in the dynamics of gap occurrence, especially in the case of the seasonality characteristic of tropical regions with a strong contrast between wet and dry seasons. In the long-term AOD-AERONET series with high temporal persistence and seasonality of the gaps, initialization based on the climatological mean provided an initial guess containing more information about the regional atmospheric dynamics, reducing distortions associated with artificial patterns introduced by simplified initialization methods.

**Fig. 4:**
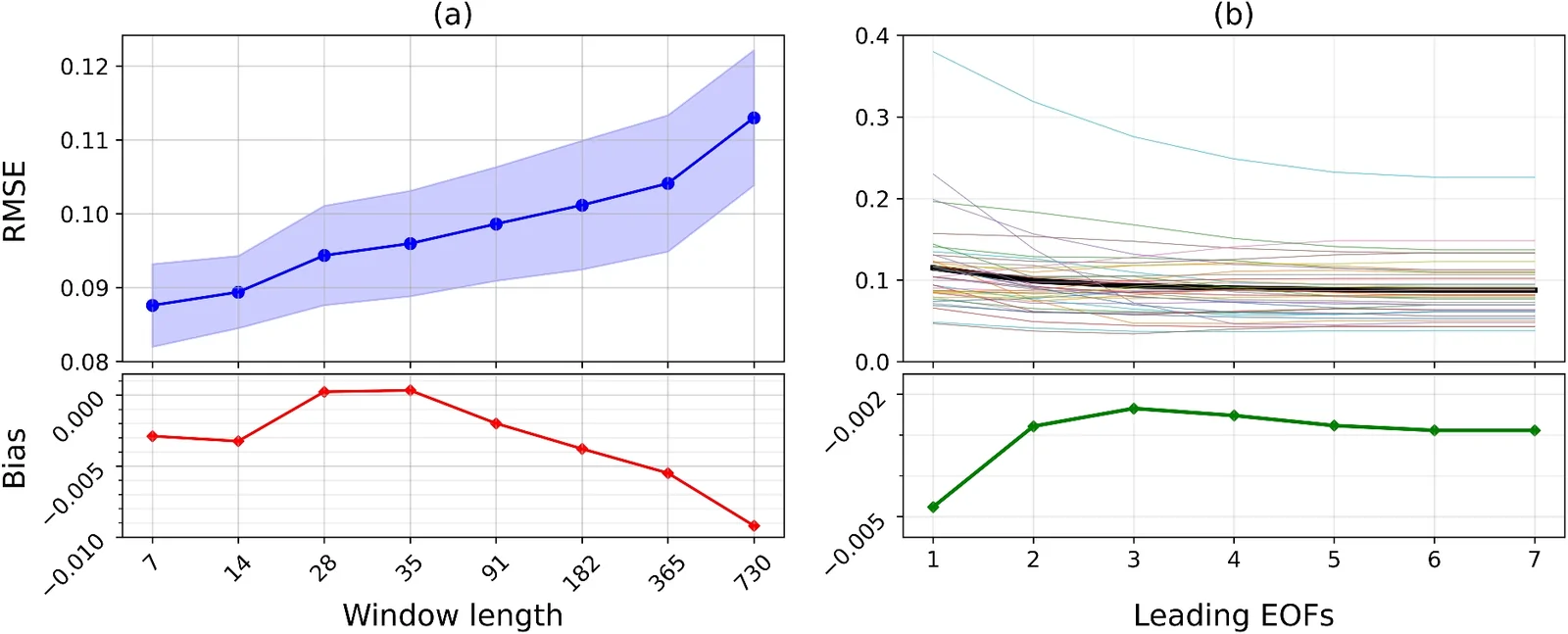
Cross-validation metrics for gap filling of the AOD-AERONET series initialized with the DOY mean. **a** Minimum RMSE value and corresponding bias for each window length. **b** RMSE and bias values for the optimal window length as a function of the number of leading EOFs used

### Iterative SSA performance and sensitivity to the adopted approaches.

#### Gap-filling with climatological initialization

The gap-filling with Iterative SSA (ISSA) initialized using the daily climatological mean, with cross-validation with random validation sets, showed the best overall performance among the two initialization strategies considered. The cross-validation metrics indicated that ISSA was able to reconstruct the incomplete AOD-AERONET series consistently with the temporal variability of the observed data, even under conditions of strong climate seasonality, systematic and persistent temporal patterns of observational gaps, and continuous intervals without valid records. Figure 4a shows the minimum cross-validation RMSE as a function of the window length *L*, together with the corresponding bias.

A general trend of increasing error was observed as *L* increased, indicating better performance for relatively short windows. The lowest RMSE was obtained for the optimal parameter combination (L,d)=(7,6), corresponding to $\text {RMSE}=0.0876$ and $\text {Bias}=0.0029$. These results indicate the high capacity of ISSA to reconstruct the temporal variability at the validation points while maintaining a low systematic deviation.

The optimal window length estimated by cross-validation partially contrasts with conventional SSA recommendations for series reconstruction, which suggest *L* values on the same order of magnitude as the series length (*n*), such as L=n/2 or L=n/3 (Golyandina, 2010; Hassani et al., 2019). However, the superior performance associated with short *L* values may be related to the intense episodic variability of the series, as larger windows tend to suppress variable frequencies and transient signals Yi and Sneeuw (2021).

The behavior of the RMSE and bias as a function of the number of EOFs used in the reconstruction (*d*), for the optimal window length, indicates a smooth and progressive reduction in error as the number of EOFs increases and the ensuing iterations that form the basis of ISSA (Fig. 4b). This suggests that a significant portion of the temporal variability was propagated to the missing points during the successive iterations. The optimal cross-validation RMSE corresponds to 35% of the mean and only 24% of the standard deviation of the observed data, indicating relatively low error values compared with the descriptive properties of the series.

**Fig. 5:**
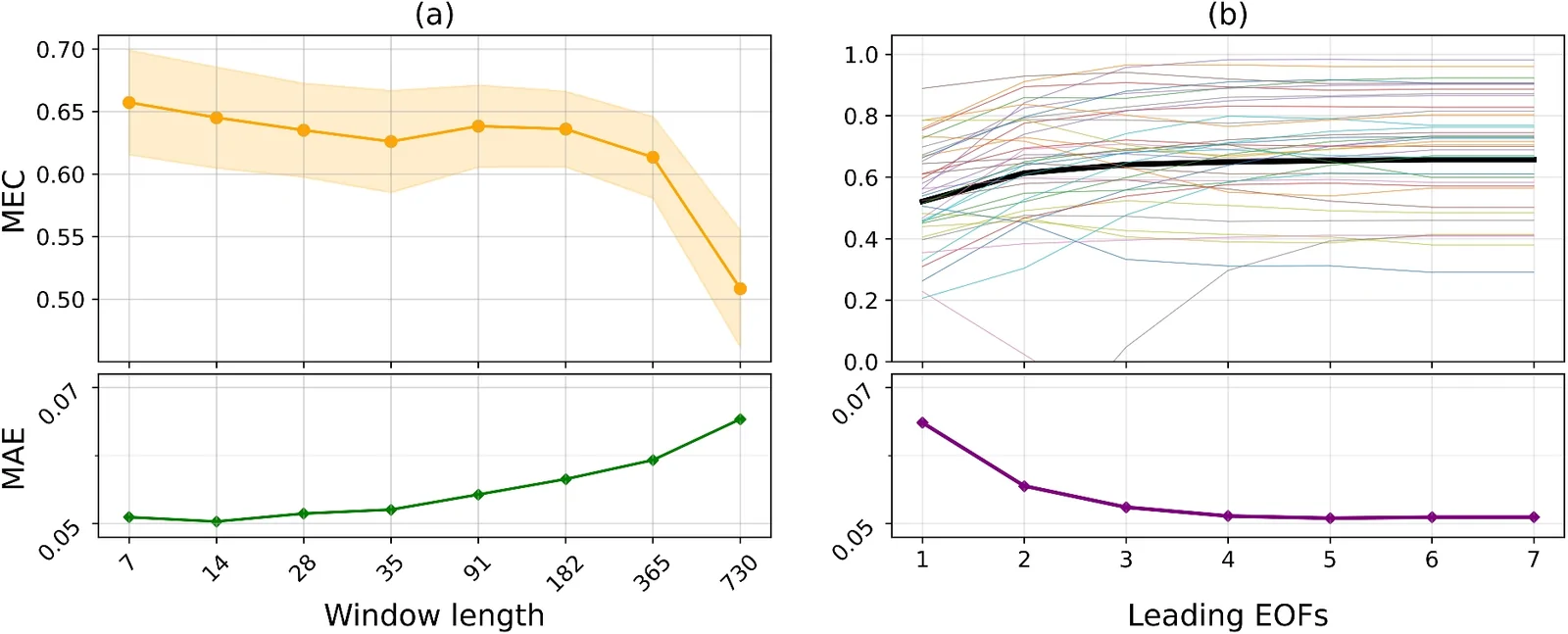
Cross-validation MEC and MAE for gap filling of the AOD-AERONET series initialized with the DOY mean. **a** Highest MEC value and corresponding MAE for each window length. **b** MEC and MAE values for the optimal window length as a function of the number of leading EOFs

**Table 1:** Comparison of the cross-validation metrics for ISSA and the corresponding DOY initialization (baseline)

Evaluation stage	RMSE	Bias	MEC	MAE
DOY-based initialization	0.1737	-0.0132	*N*/*A*	0.0952
DOY-initialized ISSA	0.0876	0.0029	0.6573	0.0509
Relative improvement$^{1}$	49.5%	$78.0\%^{2}$	*N*/*A*	46.5%

$^{1}$ For error metrics after applying ISSA

$^{2}$ Calculated using the absolute bias values

The cross-validation MEC value provides an estimate of the normalized performance improvement (Schaefli & Gupta, 2007) achieved by the ISSA gap-filling procedure relative to the initial guess based on the DOY mean. For all values of *L*, the cross-validation MEC was positive (Fig. 5a), indicating that ISSA produced reconstructions that were systematically superior to the estimates provided by the initial guess. The highest cross-validation MEC value was obtained for the optimal parameter combination (L,d)=(7,6), corresponding to $\text {MEC}=0.6573$, with an associated $\text {MAE}=0.0509$. If the parameter selection had been based exclusively on MAE, the suggested optimal window length would have been L=14 (Fig. 5a), such that the joint analysis of the metrics aided the selection of the optimal parameter combination.

The cross-validation MEC value provides an estimate of the normalized performance improvement (Schaefli & Gupta, 2007) achieved by the ISSA gap-filling procedure relative to the initial guess based on the DOY mean. The MEC value indicates that 65.73%, approximately two-thirds, of the *mean squared error* (MSE) associated with the initial climatological guess was reduced through the iterative reconstruction. This improvement indicates that ISSA was capable of recovering a significant part of the temporal structure of the series across the validation sets. This result is further supported by the RMSE, bias, and MAE values obtained under the same cross-validation setup, which were considerably lower after application of the ISSA gap-filling procedure than those of the DOY-based initialization (Table 1). Although the climatological mean reproduced part of the annual seasonality already at the initialization stage, applying iterative SSA to this initial guess yielded more accurate results.

**Fig. 6:**
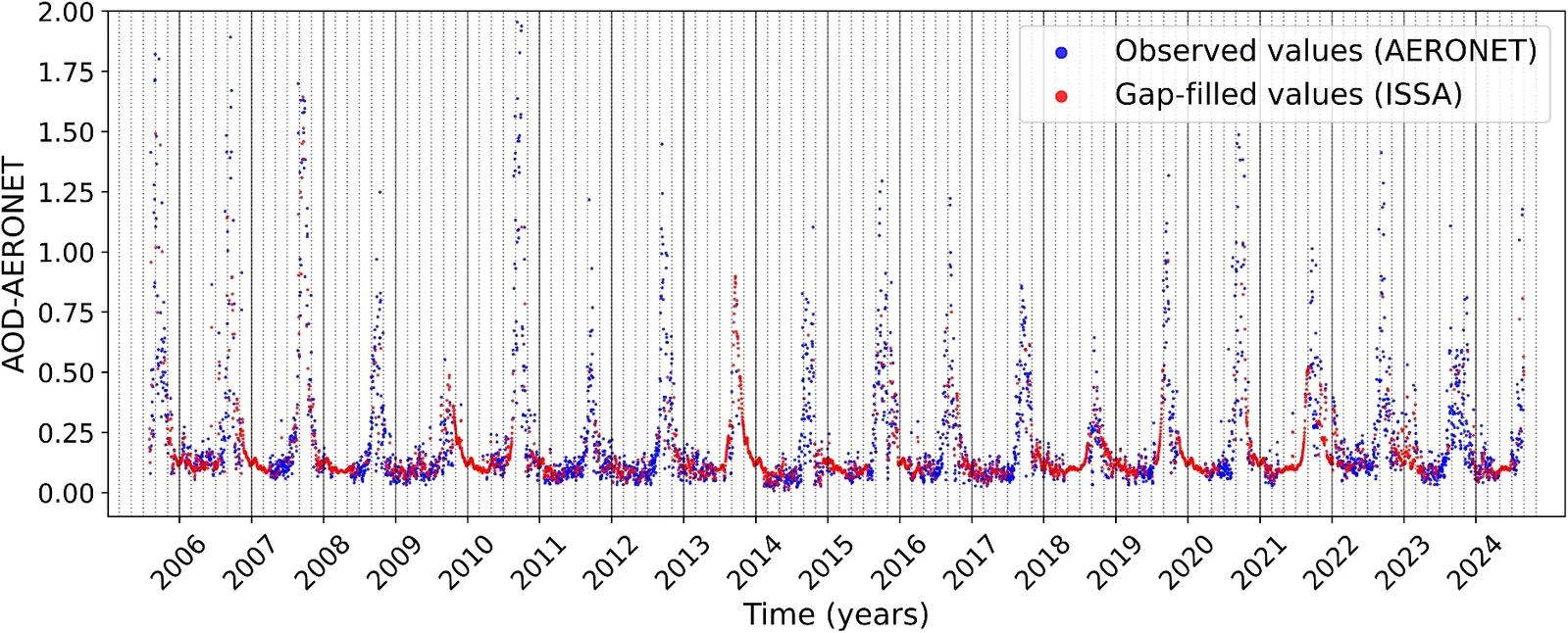
Final long-term AOD time series gap-filled by ISSA using daily climatological mean initialization and cross-validation with random observational sets. AOD values greater than 2.0 were clipped for better visualization

**Fig. 7:**
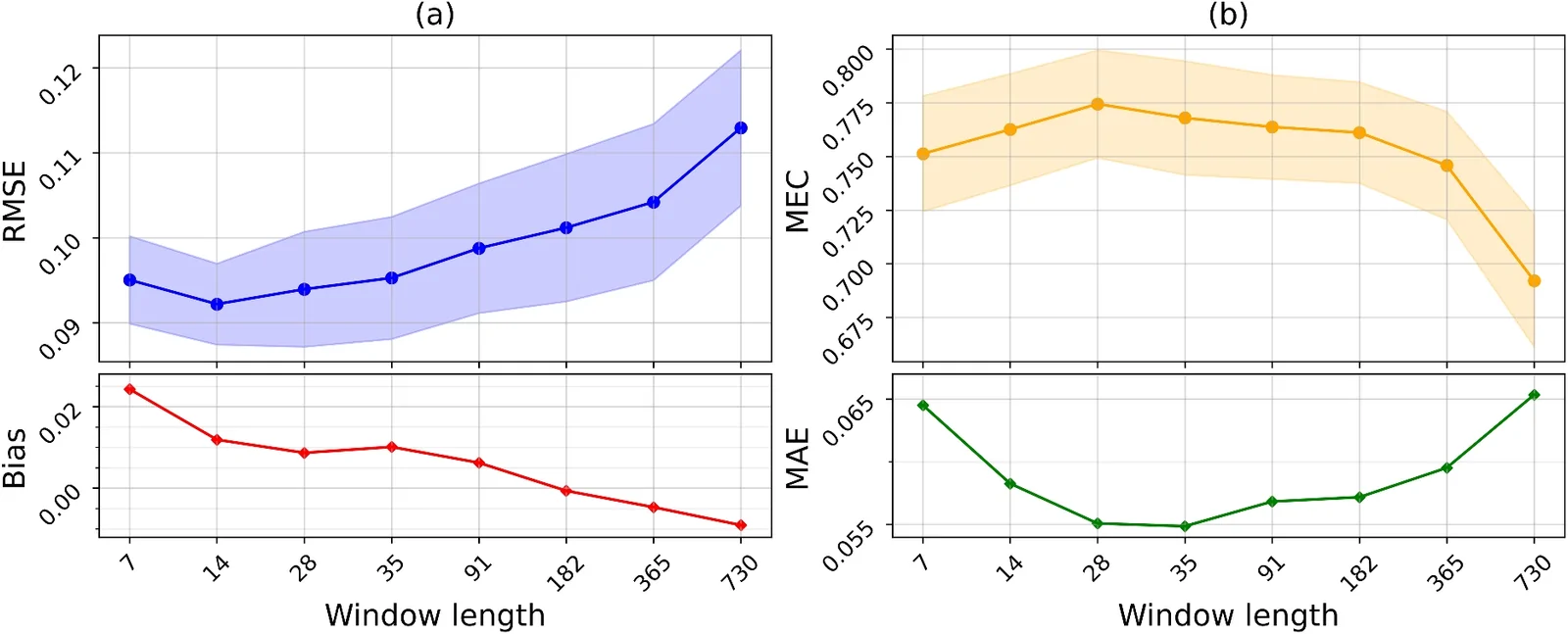
Performance metrics for ISSA with global mean initialization, evaluated using random points. **a** Minimum RMSE values and corresponding bias and **b** maximum MEC values and corresponding MAE for each *L*

For ISSA, the accuracy metrics, RMSE and MAE, exhibited a nearly monotonic increase in error with increasing tested window lengths, while improving as the number of EOFs increased for the optimal window length. The same pattern was observed for the relative efficiency (MEC). Although MEC reached negative values for certain specific parameter combinations and in a small fraction of the experiments (Fig. 5b), indicating reconstructions that performed worse than the initial guess, the overall and average behavior of the metrics demonstrates the robustness of ISSA for gap-filling the AOD-AERONET series. Collectively, the results indicate that the use of climatological initializations combined with short window lengths favors the preservation of the observed temporal variability in incomplete atmospheric series under strong seasonal influence.

The final gap-filling reconstruction was based on the optimal parameters selected through cross-validation, as required for the application of ISSA. For the climatological initialization, the resulting gap-filled series is presented in Fig. 6.

#### Global mean initialization under two cross-validation approaches.

Gap-filling using initialization with the global mean calculated from the valid observations in the series showed inferior performance compared with initialization based on the daily climatological mean in both cross-validation approaches evaluated, using random-point sets and continuous gaps.

For the random-point approach, the behavior of RMSE and its associated bias, as well as MEC and its associated MAE, for different window lengths is presented in Fig. 7.

Unlike the gap-filling initialized by the climatological mean, the best metric values were obtained for intermediate window lengths. The behavior of RMSE, bias, MEC, and MAE values pointed to different possibilities for optimal parameter combinations, which are summarized in Table 2.

**Table 2:** Optimal RMSE and MEC for three *L* values, with corresponding bias and MAE and respective *d*

Window length	RMSE (*d*)	Bias	MEC (*d*)	MAE
L=14	0.0922(13)	0.0119	0.7626(6)	0.0583
L=28	0.0939(10)	0.0087	0.7744(10)	0.0551
L=35	0.0953(7)	0.0101	0.7679(12)	0.0549

**Fig. 8:**
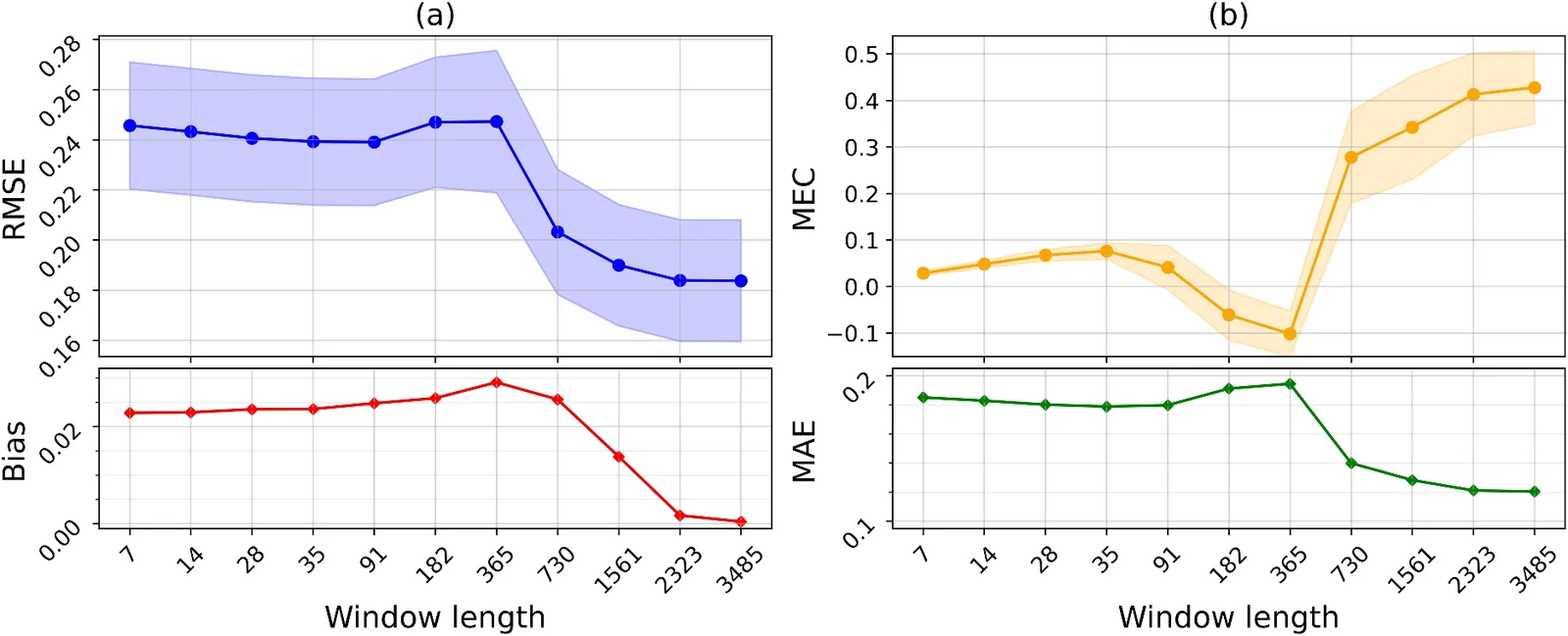
Metrics for ISSA with global mean initialization, evaluated over continuous intervals. **a** Minimum RMSE values and corresponding bias and **b** maximum MEC and corresponding MAE for each *L*

**Fig. 9:**
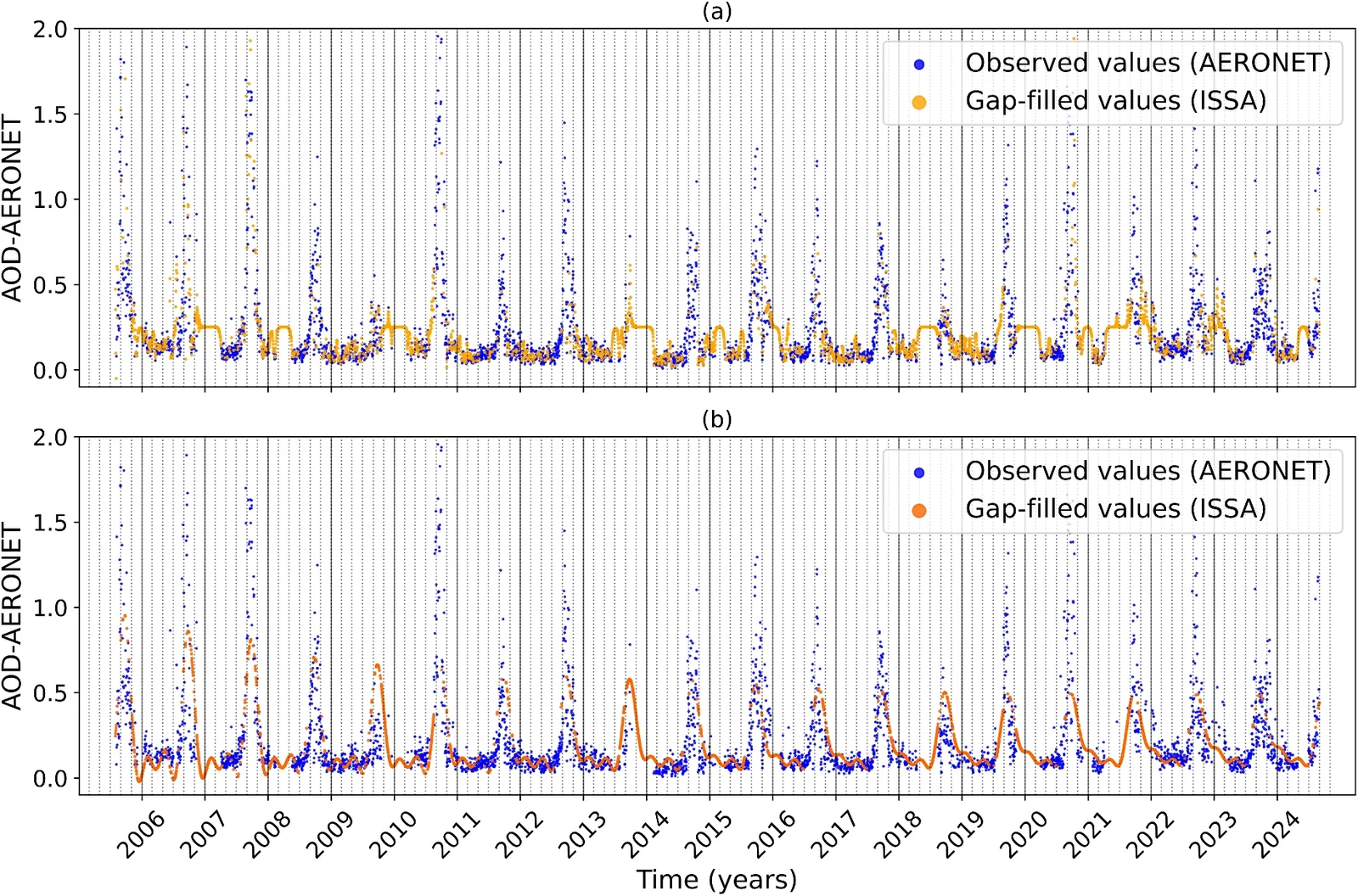
Final long-term gap-filling using ISSA with the optimal parameter combinations and global-mean initialization, evaluated through cross-validation using random points (**a**) and continuous gaps (**b**). AOD values above 2.0 were clipped

Based on the joint analysis, the parameter combination (L,d)=(28,10) provided a set of favorable metric values and was selected for the final gap-filling (Fig. 9). The RMSE and MAE metrics indicate a marginal decline in performance compared to the climatological mean initialization. However, the cross-validation MEC indicated that ISSA can produce gap-filled series that outperform the global mean initial guess across the validation experiments. The resulting MEC was higher than that obtained with the climatological mean initialization; however, because it was calculated relative to a different reference that provides less information about variability, this does not imply that the global mean approach is superior (Schaefli & Gupta, 2007; Williams, 2025).

In the other ISSA cross-validation approach based on global mean initialization, error evaluation using sets of temporally continuous gaps was proposed to address the difficulties imposed by persistent observational gaps. As a result, the behavior of the cross-validation metrics changed substantially compared with the evaluation using random sets (Fig. 8), suggesting the use of larger window lengths, which tend to better capture low-frequency variations at the expense of local patterns, such as those introduced by the global mean initialization.

In this approach, the minimum RMSE for each *L* (Fig. 8a) exhibits an approximately constant plateau up to L=365, corresponding to the annual period. From this window length onward, the RMSE decreases and stabilizes for the largest tested *L* values (*n*/3 and *n*/2), where it reaches its lowest values, practically eliminating the associated global bias. Analogously, MEC (Fig. 8b) stabilizes at higher values for larger window lengths and reaches its worst values at intermediate lengths, particularly L=182 and L=365, with $\text {MEC}<0$, indicating inferior performance compared with simple substitution by the global mean. The MAE associated with the maximum MEC under each tested *L* exhibits a behavior similar to that of the RMSE.

The optimal parameter combination for this ISSA performance evaluation was defined as (L,d)=(3485,7). This window length, equivalent to (n-1)/2, is the maximum recommended for time series reconstruction via SSA, given that $$\boldsymbol{M}_{L\times K}=\boldsymbol{M}_{L'\times K'}^T$$, where $$\boldsymbol{M}$$ is the trajectory matrix of the time series and $L^{\prime }=\left(n-L+1\right)$. The visual analysis of the final gap-filled series using global mean initialization, presented in Fig. 9, reveals significant differences from the climatological initialization approach and highlights the importance of the cross-validation scheme in representing different conditions.

The application of ISSA with global mean initialization, evaluated using random points, resulted in pronounced artificial patterns during gaps associated with persistent missing observations. Despite ISSA’s capability to fill random and scattered gaps as well as long continuous intervals, the method produces unrealistic values with spurious variations when the gap distribution follows a seasonal or systematic pattern (Musial et al., 2011), as observed in the AOD–AERONET series.

The final reconstruction with a larger window length, as suggested by cross-validation over continuous gaps, overcame the unrealistic patterns associated with global-mean initialization but introduced smoothing of variability within the gaps and instabilities at the beginning of the series.

Collectively, these results indicate that the temporal structure of both the phenomenon’s variability and the available observations exerts a significant influence on ISSA performance. In real-world application scenarios, this constitutes a significant limitation, as observations within persistent gap intervals cannot be evaluated. These results reinforce the importance of climatologically consistent initialization strategies for filling incomplete atmospheric records in tropical regions, as well as the need for evaluation procedures that more adequately represent local characteristics.

**Fig. 10:**
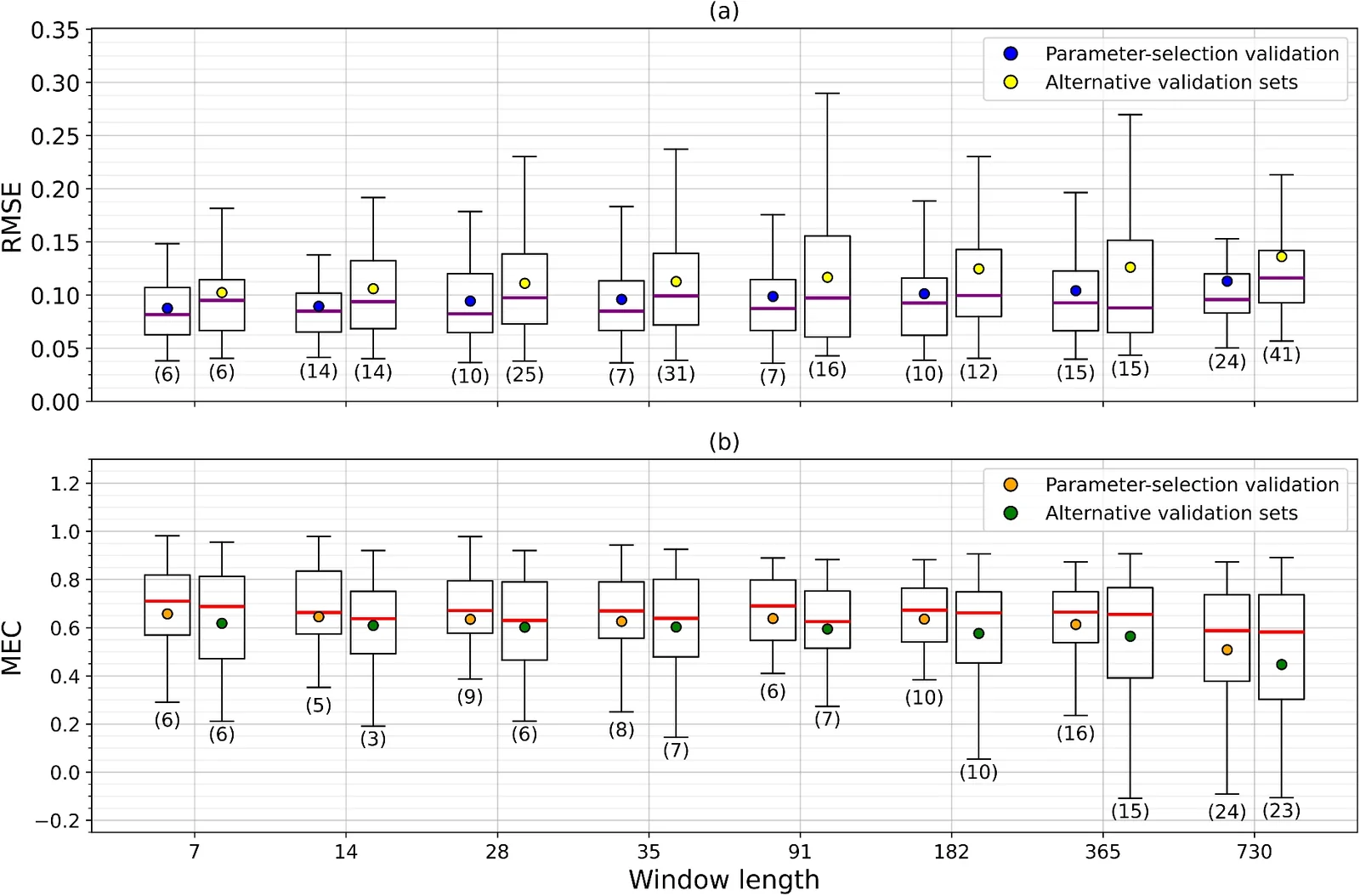
Box plots of RMSE (**a**) and MEC (**b**) from the 40 selection experiments and the 40 comparison experiments, paired by window length and considering the corresponding optimal *d* value, presented below each box plot. The circle represents the final metric for each parameter combination

#### Evaluation of the consistency of cross-validation

The cross-validation process is an inherent component of ISSA when applied to real-world gap-filling problems in environmental time series. Given the importance of this step, in addition to the analysis based on two strategies for constructing the validation sets, a comparative analysis was conducted to evaluate the behavior of the metrics. Thus, the iterative SSA gap-filling reconstruction process was repeated using a *comparison sample* of validation sets. This sample was independently generated from the same population and according to the same specifications as the *parameter selection sample* used in the reconstructions, specifically for the analysis presented in “Gap-filling with climatological initialization,” which is the focus of this comparison.

Considering RMSE as an accuracy metric and MEC as a relative efficiency metric, Fig. 10 presents the pattern of these metrics for each window length *L* and the corresponding value of *d*. The metrics were calculated as the mean across the reconstruction experiments performed with each validation set, for both the selection and comparison samples. These mean values, which correspond to the final cross-validation metrics, are shown together with box plots illustrating the distribution of the values obtained in the corresponding experiments for each specified parameter combination.

An evident feature of this comparison is the systematic difference between the metric values obtained with the selection and comparison samples, with higher RMSE values and lower MEC values for all tested values of *L*. Thus, the metrics indicate a poorer performance of ISSA in the cross-validation based on the comparison sample. The shaded regions around the final metric in Figs. 4 and 5 represent a variation interval, in both negative and positive directions, extending one unit of the *sample standard deviation of the mean*, $s({\overline{RMSE}})$ and $s({\overline{MEC}})$, of the corresponding metric. For RMSE, L=14 and L=365 represent the smallest and largest deviations from the sample mean, respectively, for which the deviation of the comparison metrics corresponds to $3.37s({\overline{RMSE}})$ and $2.39s({\overline{RMSE}})$ above the RMSE estimated from the selection sample. Similarly, for MEC, the window lengths L=182 and L=730 correspond to deviations of $1.98s({\overline{MEC}})$ and $1.31s({\overline{MEC}})$ below the below the MEC from the selection sample.

The systematic and pronounced differences between the metrics obtained from the selection and comparison samples are consistent with their independent construction, while also showing that their variation patterns with respect to *L* are practically the same. RMSE increases monotonically with increasing *L* in both cases, whereas, in general, MEC shows the opposite dependence on *L*. In addition to the similarity between the patterns obtained from the two samples, the analysis of both metrics using the comparison sample resulted in the selection of the same optimal parameter combination used in the gap-filling reconstruction presented in Fig. 6, namely (7, 6), for which $\text {RMSE}=0.1023$ and $\text {MEC}=0.6184$, corresponding to $2.6s(\overline{RMSE})$ above the RMSE and $0.93s(\overline{MEC})$ below the MEC estimated from the selection sample.

The number of EOFs used in the reconstruction, *d*, depends on the window length and obeys the inequality d≤L. In the context of Iterative SSA gap-filling, its value also represents the number of iterations. As shown in Fig. 10, for some values of *L*, the optimal number of EOFs was very close, or even identical, between the two runs based on the independent validation samples for each of the metrics considered. In particular, the optimal combination (7, 6) was the only one that was repeated for both samples and both metrics. On the other hand, window lengths of L=28, L=35, L=91 and L=730 resulted in substantially different optimal values of *d* for at least one of the two metrics analyzed.

Although the runs based on independent validation sam-ples produced numerically distinct metrics, the effects of *L* and the corresponding optimal value of *d* resulted in very similar patterns, even leading to the selection of the same optimal parameter combination, which is the objective of the cross-validation stage, and consequently to the same final reconstruction. A more in-depth assessment of the stability of the results could consider repeating the procedure with a larger number of independent cross-validation samples and under different configurations of the validation sets, such as those formed by fully random points without delimiting the population, interval-based sets, or other configurations.

### Parameter sensitivity under different approaches

The choice of parameters plays a fundamental role in the application of SSA for time series reconstruction or gap-filling, as they are related to the method’s ability to capture and distinguish information associated with trends, periodicities of different frequencies, and noise (Golyandina, 2010; Kondrashov & Ghil, 2006; Yang & Buu, 2025). The joint analysis of the tests revealed high sensitivity of the cross-validation metrics in the ISSA application to the window length *L* and the number of EOFs *d* retained in the iterative reconstruction. This dependency of the estimated error on the window length was evident both with the use of the climatological mean initialization strategy and the global mean strategy; in the latter case, the cross-validation approach also resulted in a strong sensitivity of the metrics to the window length.

The window length is intrinsically linked to the maximum rank of the trajectory matrix; consequently, higher *L* values typically allow for more EOFs to be used in the series decomposition, which, in the context of ISSA, facilitates a broader range of iterative reconstructions. Nevertheless, this relationship was not consistently observed in the conducted gap-fillings, especially regarding the results summarized in Table 2, demonstrating that the optimal value of *d* is highly dependent on the window’s capacity to effectively represent the dominant temporal structure of the series. The behavior of the performance metrics as a function of *d*, considering the optimal window for each scenario, exhibits a trend of rapid improvement for the initial *d* values, followed by a smooth variation and a subsequent performance decline for higher *d* values; this pattern underscores a clear dependency of the resulting cross-validation metrics on this parameter.

The analyzed cases suggest that the optimal cross-validation value of *L* is strongly related to the quality of the estimate provided by the initial guess, which, at one extreme, can reproduce important characteristics of the seasonality of the series and, at the other, create unrealistic patterns that can be identified by the EOFs as part of the signal. At this point, adapting the cross-validation strategy to the gap configuration and the variability characteristics of the series proved to be a relevant strategy to incorporate into the ISSA application.

The predominance of smaller optimal window lengths in the gap-filling with climatological initialization was obtained through the evaluation of cross-validation metrics over months with a high rate of observational gaps, which correspond to the wet season. During this period, the temporal variability of the AOD-AERONET series is strongly influenced by smaller-scale temporal structures related to urban emissions and the removal of particulate matter by rainfall (Franco et al., 2024; Júnior et al., 2025). Thus, the metric evaluation in this cross-validation design occurs during a period when part of the underlying dynamics of high-frequency AOD variation is strictly related to local precipitation dynamics, which may be partially suppressed by the signal smoothing resulting from the use of larger window lengths. Although larger windows are frequently recommended in classical SSA applications focused on extracting long-term trends and seasonalities (Golyandina, 2010), the results obtained in this study suggest that highly seasonal tropical atmospheric series may demand distinct parameterization strategies.

The results indicate that the ISSA parameterization is related to the physical and climatological characteristics of the analyzed atmospheric variable, including those that influence the occurrence and temporal structure of the missing observations, which are central to the ISSA application. In observational series with strong seasonality, high gap persistence, and intense episodic variability, data-driven gap-filling strategies–incorporating climatologically consistent initializations and a cross-validation design tailored to the case under study–can help preserve the observed temporal structure and improve the robustness of the iterative reconstruction.

### Final gap-fillings of the incomplete AOD-AERONET time series

The final gap-filled reconstructions of the AOD-AERONET series using the optimal parameters obtained by cross-validation showed significant differences between the evaluated initialization strategies (Figs. 6 and 9). Overall, ISSA was capable of preserving a significant part of the observed seasonality and the variability associated with high aerosol concentration events across the different parameter combinations evaluated, while reproducing these patterns at the gap-filled points.

The reconstruction initialized by the daily climatological mean presented better temporal consistency and greater visual compatibility with the climatological pattern of AOD in the study region. As observed in Fig. 6, the reconstructed values appropriately followed the seasonal alternation between periods of low aerosol concentration during the wet season and episodes of high AOD associated with the dry season and regional biomass burning. Furthermore, the reconstruction partially preserved the local patterns of observed variation at different times of the year, reducing the occurrence of artificial patterns in continuous observational gaps.

On the other hand, the reconstruction based on the global mean reproduced the annual seasonality characteristic of the underlying dynamical processes but presented some issues depending on the evaluation approach. In the random-point validation approach, the optimal parameter combination effectively reproduced the trend of higher AOD values during dry periods. However, it maintained unrealistic patterns introduced by the initialization during gaps associated with persistent cloudiness in the wet season (Fig. 9a). In the continuous-gap validation approach, the optimal parameters eliminated these unrealistic patterns during the rainy season; however, pronounced smoothing over the gaps reduced the ability to reproduce the highest AOD values associated with increased biomass-burning aerosol loading during the dry season, possibly because of the small number of EOFs retained in the final iteration (Fig. 9b). This approach also generated amplitude oscillations inconsistent with the observations at the beginning of the series, leading to physically unrealistic negative AOD values. Such fluctuations are inherent to SSA and are related to its diagonal-averaging step (Golyandina, 2020; Yi & Sneeuw, 2021).

This indicates that a window length on the order of 1 month has some capacity to preserve local events, including the unrealistic patterns introduced through the use of the global mean initialization strategy, which is a characteristic of this long-term AOD series in a tropical region. In contrast, the window length equivalent to half the series length was able to capture the dominant seasonality, including during wet seasons, bypassing the initialization patterns at the expense of local variations, particularly evident during periods of greatest AOD variation.

The climatological mean approach used information contained within the series itself, consistent with SSA-based methods. In shorter series, the DOY mean may become infeasible, and the adoption of another temporal resolution that covers all observational gaps, such as the mean calculated for each individual calendar month or the monthly climatological mean, may provide an alternative that avoids creating a systematic pattern. Such an approach can also be used as a way to remove seasonality by subtracting climatological means (Ghil et al., 2002). In the case of series with high variability, seasonality, and the systematic occurrence of single missing values or gaps, avoiding patterns that could be interpreted as natural by the EOFs–by using information available in the data themselves during the initial stages–proved to be an important strategy.

## Conclusion

This study evaluated the performance of Iterative Singular Spectrum Analysis (ISSA) in the reconstruction of a long-term AOD-AERONET time series from the Central-West region of Brazil, characterized by high climatic seasonality and a high proportion of observational gaps associated with persistent cloudiness. The results demonstrated that ISSA, when associated with climatological mean initialization, exhibited consistent performance in the recovery of the series’ temporal continuity, preserving seasonal patterns and a significant part of the variability associated with high aerosol concentration events during the dry season.

The primary natural condition associated with observational gaps in the AOD-AERONET time series has a predominantly seasonal character, and the analysis of different initialization strategies showed that the gap-filling reconstruction depends strongly on the climatological representativeness of the initial guesses used in the iterative process. Initializations based on climatological means calculated by DOY produced reconstructions that were more consistent and physically compatible with the observed seasonality of AOD-AERONET when compared to global mean initialization. Although the latter reproduced the most evident annual variability and maintained the natural dry season peaks, it resulted in gap-filling reconstructions with unrealistic patterns over long gaps or at the beginning of the series.

The data evidenced that observational gaps in AERONET site series in tropical regions exhibit a strongly non-random behavior, controlled by the regional hydrological regime and the seasonal persistence of cloudiness. The results showed that this characteristic, combined with the series length, the high proportion of gaps, and the high variability of AOD-AERONET, increases the complexity of gap-filling reconstructions and reinforces the high sensitivity of the results to the capacity of the initialization to incorporate the intrinsic temporal structure of the analyzed atmospheric variable over the gaps, especially when they occur systematically and form continuous-gap segments. The optimal parameters exhibited strong sensitivity to the cross-validation design, related to its ability to estimate statistical metrics over actual gaps, but were consistent when the method was reproduced using an independent cross-validation sample.

As limitations of this study, we highlight the fact that the evaluation was conducted on only one AERONET station located in a tropical environment with strong climatic seasonality and AOD variability. Therefore, the generalization of the results and discussions to other regions with distinct climatic regimes should be approached with caution, as variability characteristics and the formation of observational gaps follow the local hydrological regime. In the wet season, precipitation and cloudiness are associated with low AOD values and the persistence of observational gaps, whereas in the dry season, atmospheric particulate matter remains in suspension for longer periods, with a substantial increase due to biomass burning emissions, resulting in AOD peaks but a low gap rate. Furthermore, in-depth analyses of the stability of the results under different cross-validation and initialization configurations were not conducted to assess the robustness of the method, nor were performance comparisons with other gap-filling methods available in the literature conducted.

Taken together, the results reinforce that ISSA represents a promising approach for the temporal gap-filling of incomplete long-term atmospheric records with a high proportion of observational gaps and a non-random gap distribution under conditions of strong climatic seasonality, especially when associated with initialization strategies capable of preserving the climatological structure of the analyzed variable. Its performance, however, depends not only on the temporal structure of the variable but also on the temporal structure of the observational gaps used for cross-validation, such that the determination of optimal parameters must explicitly consider the pattern of gap occurrence, especially when associated with the natural characteristics of the phenomenon. Future studies may explore applications related to environmental monitoring by integrating ISSA with other statistical approaches or machine learning techniques, enhancing cross-validation processes, and evaluating the method’s performance across different atmospheric variables, temporal scales, and initialization strategies. Studies focused on assessing the stability of the method could also be conducted using complete time series with controlled insertion of gaps, allowing the quality of the final reconstruction to be objectively evaluated, rather than relying only on cross-validation metrics.

## Data Availability

The aerosol optical depth datasets analyzed during the current study are available from the AErosol RObotic NETwork (AERONET) database at https://aeronet.gsfc.nasa.gov/.
